# Emerging Roles of Malaysian Pharmacists in Asthma Management Amidst the COVID-19 Pandemic: A Narrative Review

**DOI:** 10.21315/mjms2023.30.4.4

**Published:** 2023-08-24

**Authors:** Lukman Nul Hakim Md Khairi, Shubashini Gnanasan

**Affiliations:** 1 Department of Clinical Pharmacy, Faculty of Pharmacy, Universiti Teknologi MARA, Selangor, Malaysia; 2 Department of Pharmacy, Hospital Sultanah Nur Zahirah, Ministry of Health Malaysia, Terengganu, Malaysia

**Keywords:** asthma, Malaysia, COVID-19, pharmacists, SARS-CoV-2

## Abstract

The arrival of COVID-19 pandemic in March 2020 adversely affected every aspect of human life, including the management of asthma. The pandemic has forced clinicians to revisit the application of high-risk aerosol-generating procedures in asthma management, including spirometry and nebuliser therapy. The use of commercial spacers with pressurised metered-dose inhalers to replace nebulisation is limited by the high cost and pandemic-induced stock unavailability of these inhalers. The need for social distancing, healthcare reserves reallocation, and scarce personal protective equipment has promote increased telemedicine uptake for patients’ asthma control and monitoring. Malaysian pharmacists have been providing long-term care of asthma through the introduction of the respiratory Medication Therapy Adherence Clinic (MTAC) to empower patients’ general health literacy, train and regularly evaluate their inhalation technique, and reinforce the importance of medication compliance. To minimise the use of unplanned healthcare resources and avoidable COVID-19 infection exposure, Malaysian pharmacists need to better support asthma self-management via increased uptake of written Asthma Action Plans (AAPs). Pharmacist-led asthma treatment step-down to attain the lowest effective dose of inhaled corticosteroids (ICS) has become increasingly relevant during the pandemic, as its prolonged use carries risk of numerous side effects and possible hospitalisation. Telepharmacy offers a promising model for exploration and an alternative to the traditional service delivery of asthma education. Despite not being authorised as vaccinators, Malaysian pharmacists hold strong positions in COVID-19 immunisation programmes for pharmacovigilance and advocacy. The pandemic demands an increased role for pharmacists within medication management to prevent patients from the stockpiling that can cause adverse effects on pharmaceutical supply chain. This review intends to summarise the impact of COVID-19 on asthma management, with a focus on the transitional roles of Malaysian pharmacists before and after the pandemic era.

## Introduction

With a prevalence ranging from 1% to 18% in various countries, asthma is considered one of the most common chronic diseases worldwide (1). In Malaysia, the National Health and Morbidity Survey 2011 found that the overall asthma prevalence increased to 6.4%—a 50% increase over the preceding 15 years (2). This increase could be attributed to a variety of lifestyle and environmental factors. Of note, only 32.6% of patients attended regular preventive healthcare appointments. Despite access to effective treatments, 68.1% of patients with asthma continued to experience asthma exacerbations, of which 25.8% of these patients had at least three such episodes in a single year (2).

Asthma affects people of all age groups including children and older adults. Poorly controlled asthma brings with it negative socioeconomic impacts due to productivity losses in the event of missed work and school days (3). Asthma-related emergency department visits and hospitalisations are costly and lead to significant overall per-patient healthcare expenditures. A local survey found that the cost of acute asthma management associated with hospital admission was more than ten times the cost of maintenance management in a suburban public hospital (4). Poor asthma control is a major determinant of urgent healthcare utilisation in Malaysia (5). It thus seems likely that applying strategies aimed to ameliorate asthma control would improve patient outcomes and reduce societal costs.

As of 17 November 2021, more than 2.5 million Malaysians had been infected with COVID-19 and 29,769 Malaysians had died (6). COVID-19 continues to adversely impact society worldwide and asthma management is no exception. Despite insufficient evidence to make considerable change to the management of asthma, these patients appear to have numerous characteristics that require specific considerations. A number of international professional bodies have responded by delivering recommendations on the management of asthma during the current pandemic (7). In the context of asthma treatment, pharmacists have been long known to play a critical role within the multidisciplinary healthcare team to ensure the effective provision of long-term care prior to the pandemic era (8). The current pandemic opens up opportunities for pharmacists to strengthen their roles and venture into new areas for service expansion. Thus, in this review article, we discuss the transitional roles of pharmacists in the management of asthma pre- and post-COVID-19 pandemic. Focus was given to actual and potential contributions by these professionals in the landscape of Malaysian public healthcare institutions.

### Pathophysiology of Asthma

Asthma is heterogenous in nature, and is typically associated with airway inflammation and hyperresponsiveness, in addition to variable airflow limitation (1). The variation in symptoms and airflow obstruction is subjected to time and intensity and is induced by multiple patient-specific factors including physical exercise, weather change, allergen or respiratory infections. Some individuals with asthma may experience life-threatening exacerbations which can bring substantial socioeconomic burden to patients and society (1). The most common asthma phenotypes are allergic asthma, non-allergic asthma, adult-onset asthma, asthma with persistent airflow limitation and obesity-related asthma. These phenotypes, along with biomarkers of T cells, eosinophils, mast cells, basophils, neutrophils and lymphocytes, influence the inflammatory patterns seen in patients with asthma (9). Diagnosing asthma requires combined assessment of respiratory symptoms pattern suggestive of asthma, patient history and spirometry test (1). Asthma severity can be determined based on the treatment level needed to attain symptom and exacerbations control; this severity is described as mild, moderate or severe (10).

### Are Patients with Asthma at Elevated Risk of Acquiring COVID-19 and Severe Presentations?

As infection with SARS-CoV-2 is associated with negative outcomes such as respiratory failure and subsequent death, concerns have been raised by the medical community and general population regarding whether patients with asthma are at an elevated risk of COVID-19 infection and severe medical complications. Initial reports from Wuhan, China, showed that asthma was less prevalent among those patients who were admitted with COVID-19 compared to population levels (11). This is consistent with existing evidence listing asthma outside of the top 10 comorbidities linked with COVID-19 mortality as opposed to diabetes, obesity and cardiovascular disease (12). A systematic review of 57 studies suggested that patients with asthma are at lower risk of contracting COVID-19 compared to those with no asthma. These patients also had similar clinical outcomes upon COVID-19 infection with a non-significantly different risk for severe illness, intensive care unit admission, mechanical ventilation and death (12). Also of interest, a large cohort study using primary care records of over 17 million people in England found that patients who had recently used oral corticosteroids were more susceptible to dying of COVID-19 (13). In light of such findings, the Global Initiative for Asthma (GINA) now recommends that, during this pandemic, effective asthma therapy should be continued to ensure optimal symptom control and minimal risk of severe exacerbations and the need for oral corticosteroids (14).

In addition, some preliminary studies have reported that patients with asthma are more likely to suffer from persistent symptoms known as long COVID for up to 6 months after recovery from COVID-19 infection. Pre-existing asthma is correlated with neurological, mood and behavioural changes in adult patients, while children with asthma tend to experience fatigue, insomnia and headaches following infection with SARS-CoV-2 (15, 16). Mast cell activation syndrome has been recently recognised as increasing the likelihood of patients developing this chronic COVID-19 syndrome (17).

### How Does COVID-19 Affect Asthma Care and Management?

#### Diagnosis

Making a precise initial diagnosis of asthma is important to prevent over or under-diagnosis and to look for possible concurrent conditions with chronic obstructive pulmonary disease (COPD). Given the aforementioned recommendation by GINA, an asthma diagnostic flowchart provided by the international body entails conducting spirometry testing in addition to assessing characteristic respiratory symptoms indicative of asthma and taking a detailed patient history (1). However, the pandemic has inevitably forced clinicians to change their routine diagnostic practice, as the Association for Respiratory Technology and Physiology (ARTP) guidance states that aerosol-generating procedures (AGPs) are inclusive of all respiratory tests, particularly spirometry, should be avoided. The guidance classifies procedures as AGPs mainly due to their cough-generating potential and the general assumption that all patients may carry COVID-19 infection unknowingly (11). With a reduced capacity to perform diagnostic tests in line with ARTP recommendations, there is a higher risk of misdiagnosis, as clinicians must now depend on patient history to a greater extent.

#### Adoption of Telemedicine

The COVID-19 outbreak urgently calls for virtual care adoption by respiratory specialty clinics in place of the usual face-to-face appointments. This owes to the need for social distancing, reallocation of valuable healthcare resources and scarcity in personal protective equipment (18). Also, the virtual visit is in line with recommendations by the National Institute for Health and Care Excellence (NICE) for minimisation of face-to-face contact whenever possible, including through patient follow-ups (7).

There are multiple advantages to opting for telemedicine service during this unprecedented time, including minimising exposure of healthcare professionals to infected patients, limiting risk of exposure of patients with asthma to other potentially infected patients, and removing transportation barriers for physical visits; together, these ultimately help ensure continuity of care during pandemic (19).

The exclusive conduct of virtual visits requires patients to perform peak flow meter testing at home to detect an acute asthma exacerbation remotely, as measurements during highly symptomatic times can be compared with patients’ baseline values (20). Despite its increasing use over the past 4 years, rates of telemedicine uptake by pulmonologists are still lacking (21). The COVID-19 pandemic has inspired multiple healthcare innovations that under ‘normal’ situations would have been considered too costly and time-consuming (22). Of note, telemedicine is not excluded from reimbursement, as Medicare and other health plans have waived co-payments for telemedicine visits as a way to boost usage by healthcare providers and patients, and to facilitate its expansion (21).

#### Unfavourable Use of Nebuliser

Major international professional bodies such as NICE, the United States Centres for Disease Control and Prevention (CDC) and GINA recommend normal continuation of biologics and other asthma medications to aim for optimal symptom control during this trying time (11, 14). However, the use of short-acting beta-2 agonist (SABA) as nebuliser therapy during acute asthma requires reconsideration (23). Nebulisers can generate aerosol particles with a size range of 1 μm–5 μm, which are capable of transmitting viruses deep into the lung. The risk of transmission is unfavourably high with nebulisation, as the respiratory aerosols produced are of high volume and are able to be propelled over long distances (24).

The first community-acquired COVID-19 case in the United States was reported on 26 February 2020 and involved a hospitalised patient who received multiple AGPs including nebuliser therapy (25, 26). Therefore, GINA and local guidelines of the Malaysian Thoracic Society recommend the avoidance of nebuliser use in healthcare setting (14, 27). In contrast, guidelines from the United Kingdom advocate for continued usage of nebulisers, as it has been claimed that the aerosols produced do not contain patient-originated virus particles and that they are derived from fluid off the nebuliser chamber instead (28). However, most COVID-19 patients had mild or even no symptoms and were unaware of being infected, yet they were highly infectious (29). Also, AGP of nebulisation was found to be prone to induce cough in patients and bystanders due to the cold temperature of aerosolised medication and the larger size of the particles (30).

Alternatively, SABA for acute asthma could be delivered using a pressurised metered-dose inhaler (pMDI) and spacer, with a mouthpiece or tightly fitting face mask, if necessary (14). Prior literature shows that the use of pMDI with spacer was associated with significant economic gains (31). Another study found that pMDI with spacer was able to give similar performance to that of nebulisers in delivering SABA to paediatric patients experiencing asthma exacerbation, albeit the finding was inconclusive in adults (32).

#### Alternatives to Commercial Spacers

In various developing countries, commercial spacers with facemask are not readily accessible due to the high cost and lack of availability, particularly as the pandemic has led to a sharp increase in demand. Despite their high price, many of these therapies are not designed for re-use with other patients due to cross-infection risk (33). However, extraordinary times call for extraordinary actions. Therefore, homemade spacers created using plastic mineral water bottles, plastic soda bottles or polystyrene cups with volumes between 200 mL and 750 mL have been used as alternative options, as they are cheap, easy to make and can facilitate individual use. A systematic review found no significant difference between homemade and commercial spacers for delivering SABA to paediatric patients with asthma exacerbation (34). Homemade spacers have been adopted in Malaysian public healthcare facilities for ambulatory and inpatient care, but pharmacists must work to prepare and ensure sufficient supplies of these DIY medical devices (35). Increased use of homemade spacers during the pandemic could lead to more research to determine their efficacy in adults with asthma.

Another modality deployed during the pandemic in place of nebulisers and commercial spacers is the use of pMDI with the Venturi mask modified spacer (VMMS). A local study on its use in the Hospital Kuala Lumpur’s outpatient emergency department found that SABA delivered through VMMS appeared be equivalent to nebulisers in terms of efficacy, was well-accepted by patients and staff members, and offered a more cost-effective alternative to commercial spacers (33). Therefore, as resource can be the limiting factors for many healthcare facilities during this difficult times, homemade spacers and VMMS serve as efficient and promising options.

### Pharmacist-led Asthma Interventions: The Traditional Roles of Malaysian Pharmacists Prior to the Pandemic

Given their clinical expertise in patient education and management, pharmacists play distinctive roles in the multidisciplinary clinical teams involved in asthma care. Within an integrated healthcare system, pharmacists are responsible for educating patients on their asthma medicines, teach and regularly assess inhalation techniques, handle patients’ queries regarding medication safety and optimise treatment adherence (8). Also, due to their ongoing interaction with patients upon prescription refills, pharmacists can contribute actively to the management of patients’ asthma control, as they are well-versed with the disease and associated medications.

#### Delivery of Personalised Medication Counselling

Asthma requires well-implemented medication therapy management (MTM). This calls for an effective partnership between patients, pharmacists, physicians and other healthcare professionals to ensure optimal treatment outcomes, avoid drug-related problems and deliver safe and effective drug therapies (36). MTM was introduced in the United States in 2003 as part of the Medicare Modernization Act, with the core aims of improving medication adherence, providing drug education and identifying any adverse drug reaction (37). Based on the MTM framework developed for use in the United States, the Ministry of Health (MOH) Malaysia founded its own pharmacist-led clinical service, named the Medication Therapy Adherence Clinic (MTAC), in 2004 (38).

One of the key differences between MTAC and MTM is the former’s focus on adherence optimisation (39). This is highly relevant, as the rate of medication adherence is typically low among patients with asthma (i.e. between 30% and 70%), and this correlates with increased treatment costs and reduced quality of life (QoL) (8). Since its introduction, many MTAC types have been initiated for chronic conditions, and Respiratory MTAC for diseases of asthma and COPD was established in 2007. Pharmacists trained for the service are stationed at respiratory clinics to provide personalised medication counselling and to address concerns regarding medications from patients and healthcare professionals (40). Patients with poor symptom control, improper use of inhalers, frequent exacerbations and low medication adherence are recruited into the programme, with the goal of regular follow-up by pharmacists upon prescription refills (40). Prior research has shown that provision of Respiratory MTAC was associated with better assessment scores of asthma control and interval symptoms (41).

#### Provision of Asthma-Focused Patient Education

To ensure successful asthma management, patients with asthma must be equipped with general health literacy on their chronic condition. A prospective cohort study in the United States showed that insufficient health literacy was significantly associated with lower knowledge of asthma medication and incorrect use of pMDI techniques (42). As this in turn can lead to poor disease control and increased use of emergency services, the use of tailored asthma education programmes is highly recommended (42). This is also evident within the Respiratory MTAC programme, as patients with asthma are given an overview of their disease by pharmacists using a flipchart as a communication aid (40). Using flipcharts effectively helps to ensure delivery of consistent subject matter during every educational session with patients and bridges potential gaps in health literacy. During each session, patients and their family members will look at the relevant photos on the flipchart, while the pharmacists describe the educational content on the opposite side. The communication aid used involves employment of health literacy techniques including using pictures, laymen terms and two-way discussion to assist patients in learning and retaining information (43).

#### Training and Regular Assessment of Patients’ Inhaler Technique

Incorrect inhalation technique is the most frequently reported patient-linked factor leading to poor asthma control, hospital admission, emergency department visits and the need for oral corticosteroids course (8). A systemic review from 144 studies illustrated that improper inhalers use has shown no improvement over the past 40 years, with the latest prevalence being 31%. With pMDI, the most commonly observed errors are lack of coordination, incorrect speed and incorrect depth of inspiration. Of note, mistakes frequently observed with dry powder inhaler (DPI) have included improper preparation and lack of expiration prior to inhalation. Patient error associated with a lack of breath-holding after dose inhalation is common for both pMDI and DPI inhalers (44).

These findings point to an urgent call for a new strategy, particularly regarding patient education of inhalation technique. One approach is to equip pharmacists with effective specialised training on asthma and various inhaler devices. Reliance on solely written or verbal instructions is insufficient, as patients require additional step-by-step physical demonstration along with regular re-assessment (45). Patients may benefit from repetitive training during follow-up appointments to improve their technique and reduce errors. For instance, the Respiratory MTAC module includes an assessment form for pharmacists to score and grade patients’ inhalation technique as good, satisfactory, or poor depending on different inhaler devices (40). This permits systematic comparison of patients’ performance between each follow-up visit.

#### Enforcement of Medication Compliance

Due to their poor general health literacy and understanding of their medications, a substantial number of patients overestimates the side effect risks associated with inhaled corticosteroids (ICS). Even worse, they fail to appreciate the benefits of maintenance therapy with ICS given their relatively delayed onset of action compared to SABA (11). Pharmacists must ensure effective communication with patients to achieve optimal adherence to ICS therapy and to avoid over-dependence on bronchodilator use, as this incorrect practice correlates with negative outcomes of exacerbations and asthma-related death (46). Therefore, patient education should focus on multiple components related to not only disease overview and inhalation technique, but also to medication regimens and the importance of medication adherence.

Malaysia’s Respiratory MTAC module aptly includes a responsibility for pharmacists to conduct pharmaceutical care intervention, as well as recommendations to physicians upon each patient review in case of therapeutic ineffectiveness, side effect occurrence and non-adherence (40). Previous research has shown that patients who had their drug therapy monitored accordingly by healthcare providers tended to demonstrate significantly better adherence, with a reduced number of maintenance medication doses and hospitalisations (47).

### Asthma Management in the Post-Pandemic Era: Opportunities and Expanded Roles for Malaysian Pharmacists

In this critical time, healthcare professionals need to transform their routine practice and be adaptive to the current situation. As key stakeholders in the management of asthma, Malaysian pharmacists need to respond to the current pandemic to maintain optimal continuity of asthma care (48). This also further reflects the evolution paradigm of the profession deeper into the state of contemporary pharmacy practice due to pharmacists’ involvement in increasingly resourceful and clinical activities (49).

#### Provision of Written Asthma Action Plans

Written Asthma Action Plans (AAPs) forms a fundamental approach in the ambulatory care of asthma, and they have gained an increasing degree of importance due to the pandemic. Systematic meta-reviews have shown that as part of asthma self-management, written AAPs lead to reduced urgent care use, better asthma control, and reduced total healthcare costs (50). Also, written AAPs are associated with improved medication adherence, health-related QoL and patient satisfaction with asthma care (51, 52).

Through their ‘Interim Guidance about COVID-19 and Asthma’ publication, GINA outlined the need to ensure that all patients are equipped with written AAPs (14). This recent recommendation was made in light of the continued difficulties expected for the foreseeable future regarding the provision of healthcare services, and of the need to reduce patients’ needs for unplanned asthma care which in turn might predispose them to avoidable COVID-19 exposure in hospital settings (1). Nevertheless, existing evidence indicates that written AAPs uptake has been very low, particularly in patients presenting to emergency departments with acute asthma (53).

A local cross-sectional study reported that only 29.1% of Malaysian asthma patients in public primary care clinics owned written AAPs prior to the pandemic and only 2% of these were provided by pharmacists (54). Low uptake of written AAPs was attributed to healthcare professionals’ unawareness of their importance, inadequate time to write and educate the patients, lack of exposure and confidence in completing the written AAPs, and large variability in content and format (55, 56). Also, the use of written AAPs was not incorporated in the latest protocol of Respiratory MTAC albeit being proposed by the Malaysian Clinical Practice Guidelines (CPG) (40, 57). Thus, post-pandemic ambulatory asthma care is expected to require Malaysian pharmacists to better support asthma self-management through the active provision and advocacy of written AAPs uptake. In addition, the issue of written AAPs complexity may mandate that pharmacists and other healthcare providers consider an application of low-literacy written AAPs which are photograph- and pictogram-based instead of standard plans (58). This may ensure better delivery of asthma counselling using clear communication tools, as only a very low proportion (6.6%) of Malaysian adults was reported to have adequate health literacy according to the National Health and Morbidity Survey (59).

#### Pharmacist-Managed Asthma Treatment Step-Down Approach

ICS have been accepted in all guidelines as the appropriate initial pharmacotherapy in the management of persistent asthma. As previously indicated, even during the pandemic, major international professional guidelines have highlighted the importance of patients taking their prescribed medications, particularly ICS (11, 14). However, their high-dose and long-term use are associated with a wide array of side effects including osteoporosis, pneumonia, fractures, glaucoma, adrenal insufficiency and skin thinning (60).

Prior literature has shown that patients’ concern for ICS-related systemic adverse effects leads to suboptimal adherence, inadequate asthma control and increased need for emergency department visits or hospitalisations (61, 62). Various guidelines recommend stepping down asthma treatment to attain the lowest effective ICS dose with the smallest risk of side effects (63). Nevertheless, research has suggested that the real-world practice of using a stepping-down strategy was rather dismal. A British study discovered that despite two-thirds of patients with asthma being well-controlled during the preceding one-year period, only 6% of them underwent treatment step-down (64).[Table t1-04mjms3004_ra]

Malaysian pharmacists will need to take up an emerging role in stepping down asthma treatment post-pandemic amidst the need for reducing urgent healthcare uses associated with medication-associated adverse effects. Ensuring effective uptake of such step-down approaches requires inter-professional collaboration with pulmonologists and physicians (65). Accessibility to updated local guidelines on asthma management and stepping-down strategies will boost pharmacists’ knowledge and reassurance that they are practising appropriately (66). Thus, this pharmacist-managed asthma intervention may benefit from inclusion in the existing local protocol of Respiratory MTAC (40). Furthermore, treatment step down will need to be considered at every follow-up visit, with accompanying discussion on written AAPs and treatment goals (66). Pharmacists need to communicate thoroughly with patients about the risks and benefits of long-term and high-dose ICS usage. This can essentially assist patients in making informed decisions, managing their conditions proactively and increasing medication adherence (67).

#### Access to Asthma Management through Telepharmacy

As previously described, urgent calls for containment measures in the post-pandemic era have promoted the relevance of telemedicine. A systematic review showed that pharmacist-managed telemedicine interventions in the ambulatory setting have been delivered in various modes of communication including telephone, video consultation, text messaging, email, automated electronic reports or fax. These interventions have been shown to bring positive outcome in chronic diseases management, including in diseases such as asthma, COPD, diabetes and hypertension with regards to disease management, self-management behaviour and medication compliance (68). Research also suggests that involvement of telehealth-capable pharmacists in a community-engaging asthma educational programme improved asthma control in the majority of patients following six visits within a single year (69).

Existing evidence on the impact of telepharmacy-based models remains inconclusive, mostly attributable to the poor internal and external validity of the available studies (70). Nonetheless, amidst the global pandemic, Malaysian pharmacists through the telemedicine models have a valuable opportunity to improve access to asthma management in the ambulatory care setting for distant delivery of asthma education. Internalisation of telepharmacy in local ambulatory asthma care requires engagement of pharmacy executives, health-system administrators and other stakeholders to explore and develop suitable delivery models (70).

#### Roles with COVID-19 Vaccination

The recent availability and access to COVID-19 vaccines encompasses the evolving roles of asthma care pharmacists. As one of the most effective modern medical interventions within public health, vaccination has quickly gained relevance in bringing the pandemic to a halt (71). Although the delivery of safe and effective COVID-19 vaccines will be game-changing, the successful combat of vaccine hesitancy appears to possess an additional decisive role to ensure control of the global pandemic (72).[Table t2-04mjms3004_ra]

At present, GINA recommends COVID-19 vaccination for patients with asthma as long as they have no previous history of an immediate or severe allergic reaction to the vaccine or any of its excipients (14). Despite the fact that Malaysian pharmacists are not authorised as vaccination providers based on National Immunisation Programme regulations, they hold strong positions as advocates for vaccination (73). Functionally, pharmacists are easily accessible to patients with asthma upon ambulatory care to provide counselling on immunisation risk and benefits, dispel misinformation about the vaccine and disease, and strongly recommend COVID-19 vaccination (74). Pharmacists may also take up the opportunity to be actively involved in pharmacovigilance activities and studies assessing COVID-19 vaccine hesitancy among patients with asthma and its potential impacts in the nation (72).

#### Intensified Roles within Medication Management

The COVID-19 pandemic further highlights the role of pharmacists in medication management. Issues with global medication shortages have been in existence long before the beginning of the current pandemic. COVID-19 inevitably intensifies the magnitude of the problem due to the lockdown and expanded demand, and given that China is the main manufacturer and exporter of world’s active pharmaceutical ingredients (7). Additionally, anecdotal reports have been published on the practice of patients stockpiling their medications in response to panic buying and self-isolation preparation. This abrupt rise in demand caused a substantial and adverse effect on the pharmaceutical supply chain (48). As medication gatekeepers, pharmacists take seriously their responsibility to prevent further drug shortages due to the pandemic. Patients and the public need to be reassured about the continued availability of inhalers and other drugs for asthma. Thus, pharmacists can help emphasise to patients that there is no need for stocking up on inhalers even during these trying times. In addition, Malaysian pharmacy executives should consider developing supply policies applicable for the pandemic to avoid unneeded stockpiling by patients including maximum quantity of medication dispensed (7). This may simplify the work process and create transparency regarding the services offered to patients.

## Conclusion

In this time of global health crisis, all healthcare professionals are required to be adaptive and innovative to respond to the detrimental impact of the pandemic on disease management and pharmacists are no exception. The traditional and emerging roles of Malaysian pharmacists described in this article are non-exhaustive, as they serve to demonstrate just some of the areas in which pharmacists can both continue and expand their contributions within asthma management in response to the pandemic.

As these expanded roles are seen to open opportunities for pharmacists to improve the state of local pharmacy practices, this also seeks to uplift the professional status of pharmacists through their involvement in more resourceful and clinical activities. Nevertheless, most of these activities require structured programme planning and implementation. Thus, their success is highly dependent on active engagement by multi-level stakeholders consisting of pharmacy executives, health-system administrators, participating pharmacists and other relevant parties.

## Figures and Tables

**Table 1 t1-04mjms3004_ra:** Characteristics of articles from a literature search for the roles of pharmacists in the pre-pandemic era

No.	Author	Objectives	Study design	Population	Findings
1	Al-Tameemi and Sarriff (39)	To determine the knowledge, attitude, and practice among pharmacists in a Malaysian tertiary care centre on MTM service.	Self-administered survey	93 pharmacists	Most pharmacists have high awareness, show positive attitude about MTM service and are willing to become MTM service provider in the future. Inadequate training and the need for considerable budget are barriers for future provision of MTM service.
2	Khalid et al. (41)	To understand the roles of Respiratory MTAC in complementing management of paediatric bronchial asthma on the provision of clinical assessment, counselling, and monitoring.	Retrospective cohort study	87 outpatient paediatric bronchial asthma	Patients enrolled to Respiratory MTAC have significantly higher assessment scores for interval symptoms and asthma. The assessment of pMDI inhalation technique is significantly poor without Respiratory MTAC assessment.
3	Paasche-Orlow et al. (42)	To assess the relationship between poor health literacy and difficulties understanding and maintaining instructions about appropriate pMDI technique and discharge medications.	Prospective cohort study	73 adults admitted with a physician-diagnosed asthma exacerbation	Inadequate health literacy is significantly related to poor pMDI technique and suboptimal asthma medication knowledge. No significant association found between inadequate health literacy with difficulties understanding and maintaining instructions.
4	Sanchis et al. (44)	To determine the prevalence of errors in inhaler use over the previous four decades in patients prescribed with pMDI or DPI.	Systematic review of 144 articles	54,354 subjects who performed observed tests of inhalation technique	Inappropriate inhalation technique is frequent and shows no improvement over the past 40 years for both pMDI and DPI inhalers.
5	Bosnic-Anticevich et al. (45)	To compare the impacts of two educational interventions delivered to pMDI users in community pharmacy.	Randomised controlled parallel-group study	52 patients with asthma or COPD	Additional physical demonstration results in significant improvement of pMDI technique over time than written and verbal instructions alone.
6	Gamble et al. (47)	To assess if non-adherence to combination inhaler of ICS/long acting beta-2 agonist in difficult-to-treat asthma could be improved using (i) a simple concordance interview and (ii) a menu driven psycho-educational intervention, with superior asthma control.	Multi-level interventional study	83 subjects who were referred to a Specialist Difficult Asthma Service	Poor adherence in difficult-to-treat asthma is common, but when identified and targeted through a concordance discussion can be significantly improved and be associated with improved asthma outcomes. Simple menu driven intervention approach can further enhance adherence in this patient cohort.

**Table 2 t2-04mjms3004_ra:** Characteristics of articles from a literature search for the roles of pharmacists in the post-pandemic era

No.	Author	Objectives	Study design	Population	Findings
1	Pinnock et al. (50)	To determine if asthma self-management improves asthma control and decreases use of healthcare resources	Systematic meta-review	Patients with asthma	Asthma self-management can decrease unscheduled care, improve asthma control and quality of life, without an increase in total healthcare costs
2	Gibson et al. (51)	To analyse the effects of asthma self-management when combined with regular medical review	Systematic review of 36 RCTs	Adults with asthma	Self-management significantly reduces hospitalisations, emergency department visits, days off school or work, night-time symptoms, and quality of life
3	Patel et al. (52)	To assess the association between having written AAPs and attitudes to keep asthma in control and patient satisfaction with care	Cross-sectional analysis of baseline data from an RCT	808 women with asthma aged ≥ 18 years old	Majority received written AAPs from their physicians. Patients without written AAPs are less likely to be adherent to their asthma medication, initiate discussion about asthma during review, to have a peak flow meter, and be satisfied with their asthma care
4	Cross et al. (53)	To investigate the frequency of utilisation and contents of written AAPs in urban Canadian EDs	Secondary analysis of randomised trials	176 patients aged 17 years old to 55 years old who were treated for asthma exacerbation	Possession of written AAPs among patients presented to ED for asthma exacerbation is dismal. Patients who possess written AAPs use inappropriate strategies to abort or alleviate severity of asthma
5	Salim et al. (54)	To determine the frequency of written AAPs possession among asthma patients and their attributes in Malaysia primary care practices	Self-administered survey	550 adult patients with asthma	Only one in every three patients owns written AAPs. Written AAPs are primarily given by the doctors, as opposed to nurses and pharmacists. Patients still lack education and confidence to own and use written AAPs
6	Yin et al. (58)	To assess if physicians who used a low-literacy written AAPs demonstrate better quality of asthma counselling compared to use of standard written AAPs	Randomised controlled study	119 physicians at two academic centres	Use of low-literacy written AAPs improves the asthma counselling quality by physicians by promoting use of laymen term and highlighting key aspects of asthma care
7	Bloom and Quint (64)	To evaluate the prevalence of asthma stepping-down in England	Retrospective cohort study	67,451 asthma patients aged ≥ 18 years old	Although two-third of mild-to-moderate asthma patients were eligible for treatment de-escalation, only one-tenth were stepped down
8	Gums et al. (65)	To assess if asthma control improves in patients who receive physician-pharmacist collaborative management (PPCM) upon medical review visit	Prospective pre-post study	126 asthma patients aged ≥ 12 years old	PPCM care model reduces asthma-related ED visit and hospitalisations, and improved asthma control and quality of life
9	Niznik et al. (68)	To determine the effect of telemedicine interventions by clinical pharmacist on clinical outcomes	Systematic review of 34 studies	Clinical pharmacist services	Clinical pharmacy telemedicine intervention in the ambulatory setting is associated with a positive impact on outcomes of clinical disease management, self-management, and adherence in the management of chronic diseases
10	Brown et al. (69)	To assess the feasibility of telepharmacy services provision within a rural community pharmacy in the delivery of asthma education	Prospective pre-post study	20 asthma patients aged ≥ 5 years old	Telepharmacy services are associated with well-controlled asthma by the third educational visit and remains control for the remaining 9-months follow-up period
11	Sallam (72)	To assess the global acceptance rates towards COVID-19 vaccination	Systematic review of 30 studies	General public	Suboptimal rates of COVID-19 vaccination acceptance were observed in the Middle East, Africa, Russia and some European countries
